# Molecular analysis of the dual targeting of the epidermal growth factor receptor and the O^6^-methylguanine-DNA methyltransferase with a double arm hybrid molecule

**DOI:** 10.18632/oncotarget.25120

**Published:** 2018-10-12

**Authors:** Martin Rupp, Zhor Senhaji Mouhri, Christopher Williams, Bertrand J. Jean-Claude

**Affiliations:** ^1^ Cancer Drug Research Laboratory, Department of Medicine, Division of Medical Oncology, The Research Institute of the McGill University Health Center/Glen Hospital, Montreal, QC, H4A 3J1, Canada; ^2^ Scientific Support, Chemical Computing Group Inc., Montreal, QC, H3A 2R7, Canada

**Keywords:** EGFR, MGMT, combi-molecule, kinase inhibition, combination chemotherapy

## Abstract

Disordered expression of the epidermal growth factor receptor (EGFR) has been associated with induction of DNA repair genes (e.g. XRCC1, ERCC1) and resistance to radiation and genotoxic drugs. However, our previous work showed that EGFR inhibition did not affect O^6^-methylguanine-DNA methyltransferase (MGMT)-mediated resistance. In order to block uncoupled events associated with EGFR and MGMT, we designed MR30, a single molecule termed “combi-molecule” that contains a quinazoline arm targeted to EGFR and an O^6^-benzylguanine (O^6^-BG) moiety to block MGMT. Molecular analysis of the mechanism of action of its two arms showed that: (a) it could block EGFR phosphorylation, (b) down-regulate the RAF-MAPK and the PI3K-AKT pathways, and (c) covalently modify MGMT through S-benzylation, as confirmed by MALDI analysis of a direct binding assay with isolated MGMT, (d) it induced a dose-dependent down-regulation of MGMT in lung and melanoma cells. The pleiotropic mechanism of action of MR30 culminated into strong growth inhibition (IC_50_: 0.018-6.02 μM), with superior activity when compared with an equimolar combination of gefitinib (a clinical EGFR inhibitor) and O^6^-BG (a known MGMT inhibitor). Pulse exposure experiments were required to attenuate the contribution of EGFR inhibition to the strong potency of MR30, thereby allowing to achieve the dose level required to sensitize cells to temozolomide (TMZ). Indeed, MR30 significantly sensitized EGFR-MGMT co-expressing cells to TMZ (p<0.05-0.0001). The results in toto suggest that MR30 is the first prototype of agents that may be used against tumours addicted to EGFR and to sensitize resistant tumours co-expressing EGFR and MGMT to TMZ.

## INTRODUCTION

Disordered expression of receptor tyrosine kinases has been associated with resistance to radiation and DNA damaging drugs such as cisplatin and temozolomide (TMZ), a clinical alkylating agent that induces O^6^-methyl, N^7^-methylguanine and N^3^-methyladenine lesions [1–7]. While N^7^-methylation alters the hydrogen bonding patterns of guanine in duplex DNA and N^3^-methyladenine is known to induce promutagenic lesions, the O^6^-methylguanine remains the most cytotoxic adduct [8–10]. EGFR is one of the most investigated receptor tyrosine kinases in the context of DNA repair. Through the mitogen-activated protein kinase (MAPK) pathway, EGFR activation leads to expression of DNA repair genes [11] and through the phosphatidylinositol-3 kinase (PI3K) pathway, it exerts an antiapoptotic effect [12]. Based on the premise that activation of EGFR leads to induction of DNA repair proteins such as X-ray repair cross-complementing protein 1 (XRCC1) and excision repair cross-complementation group 1 (ERCC1), our group embarked onto the design of drugs termed “combi-molecules” that can induce a tandem targeting of EGFR and DNA and demonstrated that indeed the latter dual targeted molecules could down-regulate XRCC1 and ERCC1 through their EGFR inhibitory arm and inducing high levels of DNA strand breaks through their DNA alkylating arm [11]. We showed that the dual targeted approach termed “combi-targeting” translated into massive cell death by apoptosis. While XRCC1 and ERCC1 play an important role in the response to radiation and cisplatin and were shown to be induced through EGFR signaling, questions remained about O^6^-methylguanine-DNA methyltransferase (MGMT), which is one of the most clinically relevant DNA repair proteins in the context of treatment of solid tumours [13, 14]. Expression of MGMT in glioblastoma or melanoma is a marker for resistance to TMZ in the clinic [13, 15]. Promoter methylation of MGMT is now clinically used in the management of glioblastoma multiforme [13]. Importantly, questions relative to the association between EGFR activation and MGMT-mediated repair has been molecularly and pharmacologically addressed by our group [12]. Using double arm combi-molecules designed to down-regulate EGFR and methylate DNA, we demonstrated that MGMT expression was uncoupled with down-regulation of EGFR signaling. Accordingly, targeting EGFR alone does not lead to down-regulation nor depletion of the function of MGMT. Therefore, here we rationally designed a molecule to block EGFR and MGMT, thereby compensating for the deleterious effects of the uncoupled mechanism of EGFR expression and MGMT expression. Such a type of molecules could be used alone in tumours addicted to EGFR or to enhance the benefit of TMZ-based chemotherapy in tumours co-expressing EGFR and MGMT.

In order to fully target the uncoupled EGFR and MGMT regulation, we use our novel combi-targeting approach, which consists of designing single molecules termed “combi-molecules” to exhibit pleiotropic effects. We have now classified our combi-molecules as type I [16–19], which are designed to be hydrolytically cleaved to induce their antitumour activity and type II, that are combi-molecules that do not require hydrolytic cleavage to induce their activity [20, 21]. Indeed, we previously showed that a single molecule designed to block EGFR and damage DNA could block EGFR phosphorylation and its downstream RAF-MAPK and PI3K-AKT pathways, induce p53 and Bax and ultimately trigger apoptosis in tumour cells. However, in a panel of cells with varied levels of MGMT, it was found that its potency was partly mitigated by MGMT expression [12]. Here, we sought to study a model whereby, a single molecule can exhibit multiple effects associated with EGFR inhibition in addition to down-regulate MGMT. Down-regulation of MGMT can be achieved on the basis of its very mechanism of action that consists of repairing the O^6^-methylguanine DNA lesion by transferring the alkyl group onto its own cysteine residue, followed by proteolytic digestion of the resultant S-alkylated protein [22–24]. O^6^-BG, the first inhibitor of MGMT [25], contains an O^6^-benzyl group that is transferred to its cysteine residue followed by subsequent digestion in a manner similar to S-methylated MGMT. [26, 27]. Thus, we designed MR30 to contain a quinazoline arm capable of targeting EGFR and an O^6^-BG-like moiety to target MGMT, with the premise that such a molecule will be able to target the EGFR-MGMT uncoupled paths by down-regulating EGFR signaling and decreasing MGMT levels. While Wanner *et al.* [28] and Sun *et al.* [29] reported the synthesis of hybrid O^6^-BG-DNA targeting molecules, this is the first report on the design and mechanism of action of a dual EGFR-MGMT targeted molecule in tumour cells.

## RESULTS

### Chemistry

**Figure 1:**
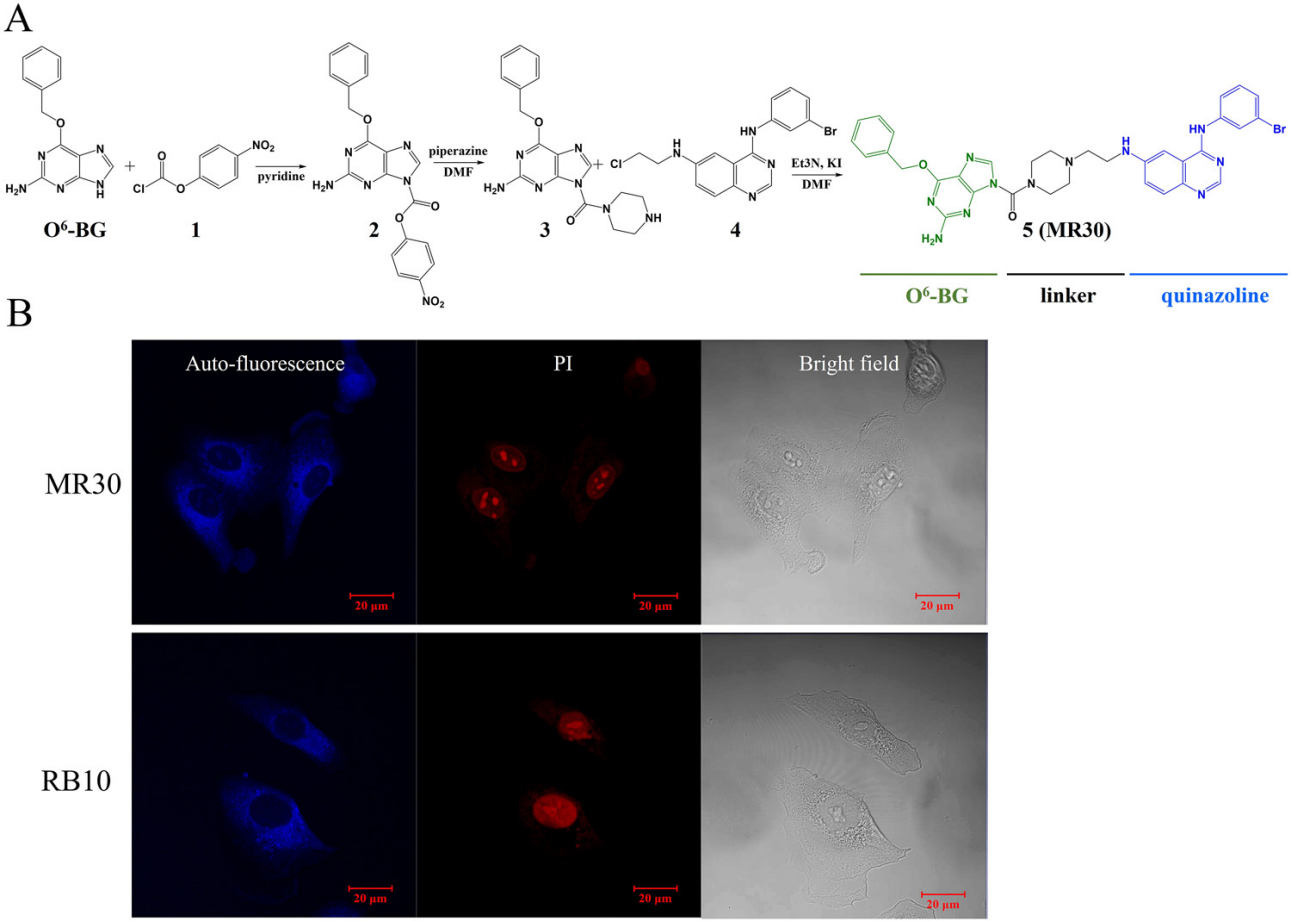
Chemistry and subcellular distribution of MR30 **(A)** Synthesis of the EGFR-MGMT targeted molecule, MR30. **(B)** Subcellular distribution of MR30 and RB10, two blue fluorescent quinazolines, in the non-small cell lung cancer cell line A549. MR30 penetrated the A549 cells and showed same distribution pattern as an experimental EGFR inhibitor, RB10.

We designed MR30 to contain a quinazoline warhead for targeting EGFR and an O^6^-BG moiety to exert MGMT inhibitory potency. For purpose of druggability, the molecule was designed with a short water soluble ethylaminopiperazine linker and the resulting mass was 694.6 g/mol. The synthesis of MR30 proceeded according to Figure 1A. Briefly, O^6^-BG was treated in pyridine with chloroformate **1** to give carbamate **2**, which was reacted in DMF with excess of piperazine to afford urea **3**. Alkylation of the piperazine ring of **3** with the chloroethylaminoquinazoline **4** gave **5** as a yellow powder (MR30). MR30 showed good water solubility when diluted from a DMSO stock solution and with ethanol 25%/cremaphone 25% in saline, a concentration as high as 7 mM could be achieved without any trace of precipitation.

### Mechanism of action

#### Cell penetration using fluorescence imaging of MR30 in A549 cells

While MR30 was designed to be a small combi-molecule with dual targeting functions, its molecular weight was greater than the 500 limit prescribed by the Lipinski's rule for druggability [30]. Thus, we tested its ability to diffuse into human cells *in vitro*. MR30 fluoresced in the blue (excitation wavelength 280 nm, λmax emission 458 nm), thereby allowing to monitor its distribution in the cells by fluorescence microscopy. MR30 exhibited good cell penetration with a preferential perinuclear distribution after 3 h drug exposure in the A549 cells. This distribution paralleled that of other molecules (e.g. RB10) previously synthesized in our laboratory that contain the aminoquinazoline warhead [31] (Figure 1B).

#### Dissection of the dual function of MR30

Having shown that MR30 can penetrate the cells, we sought to determine whether its two arms (EGFR-MGMT targeting) were exerting their respective action in whole cells. For this study, we chose the A549 and H1650 [EGFR exon 19 deletion (d746-750)], two cell lines representative of non-small cell lung carcinoma for which gefitinib is indicated [32, 33]. The cell line A375 is representative of malignant melanoma, a disease for which methylating agents of the TMZ class are clinically indicated [34]. The three cell lines co-express EGFR and MGMT.

### EGFR targeting arm

#### EGFR targeting and downstream signaling

**Figure 2:**
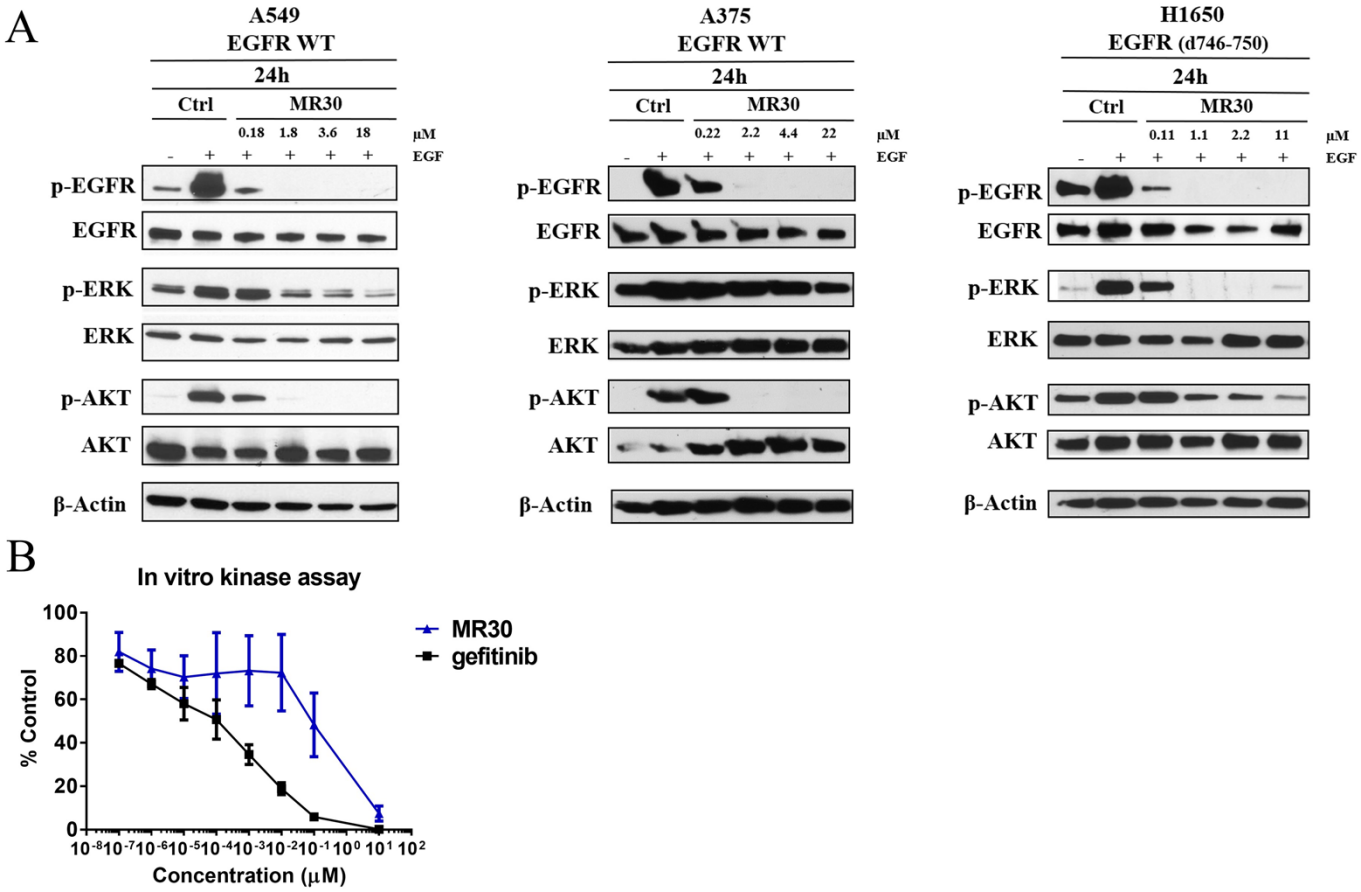
Effects of MR30 on cell signaling **(A)** Inhibition of phosphorylation of EGFR and downstream signaling proteins, p-ERK1/2 and p-AKT by MR30 was studied at the 0.1, 1, 2 and 10x IC_50_ concentrations on A549 (wild-type EGFR), A375 (wild-type EGFR) and H1650 (EGFR d746-750) by western blot. MR30 strongly inhibited EGFR autophosphorylation at submicromolar concentrations, which has led to inhibition of downstream signaling by inhibiting p-ERK1/2 and p-AKT. **(B)** MR30 and gefitinib were tested in *in vitro* EGFR kinase assay. MR30 as well as gefitinib showed dose dependent inhibition of EGFR phosphorylation. Gefitinib induced stronger inhibition of EGFR phosphorylation than MR30.

For assessing EGFR inhibitory potency, the cells were starved for 24 h and stimulated with EGF in the presence or absence of MR30 (Figure 2A). Surprisingly, MR30 despite its size could induce inhibition of EGFR autophosphorylation in whole cells at submicromolar concentrations. The results showed that MR30 induced strong blockade of EGFR autophosphorylation after 24 h. Furthermore, inhibition of EGFR phosphorylation by MR30 led to inhibition of its downstream signaling cascade by inhibiting phospho-protein ERK1/2. MR30 also induced a dose-dependent inhibition of PI3K signaling pathway as evidenced by its strong inhibition of AKT phosphorylation. Inhibition of AKT phosphorylation was observed at concentrations as low as 0.18 μM. These results are in agreement with the *in vitro* kinase assay, where gefitinib (IC_50_ < 1 nM) and MR30 (IC_50_: 0.3± 0.17 μM) induced a strong dose-dependent inhibition of EGFR phosphorylation (Figure 2B).

#### EGFR selective targeting (isolated enzyme assay)

**Figure 3:**
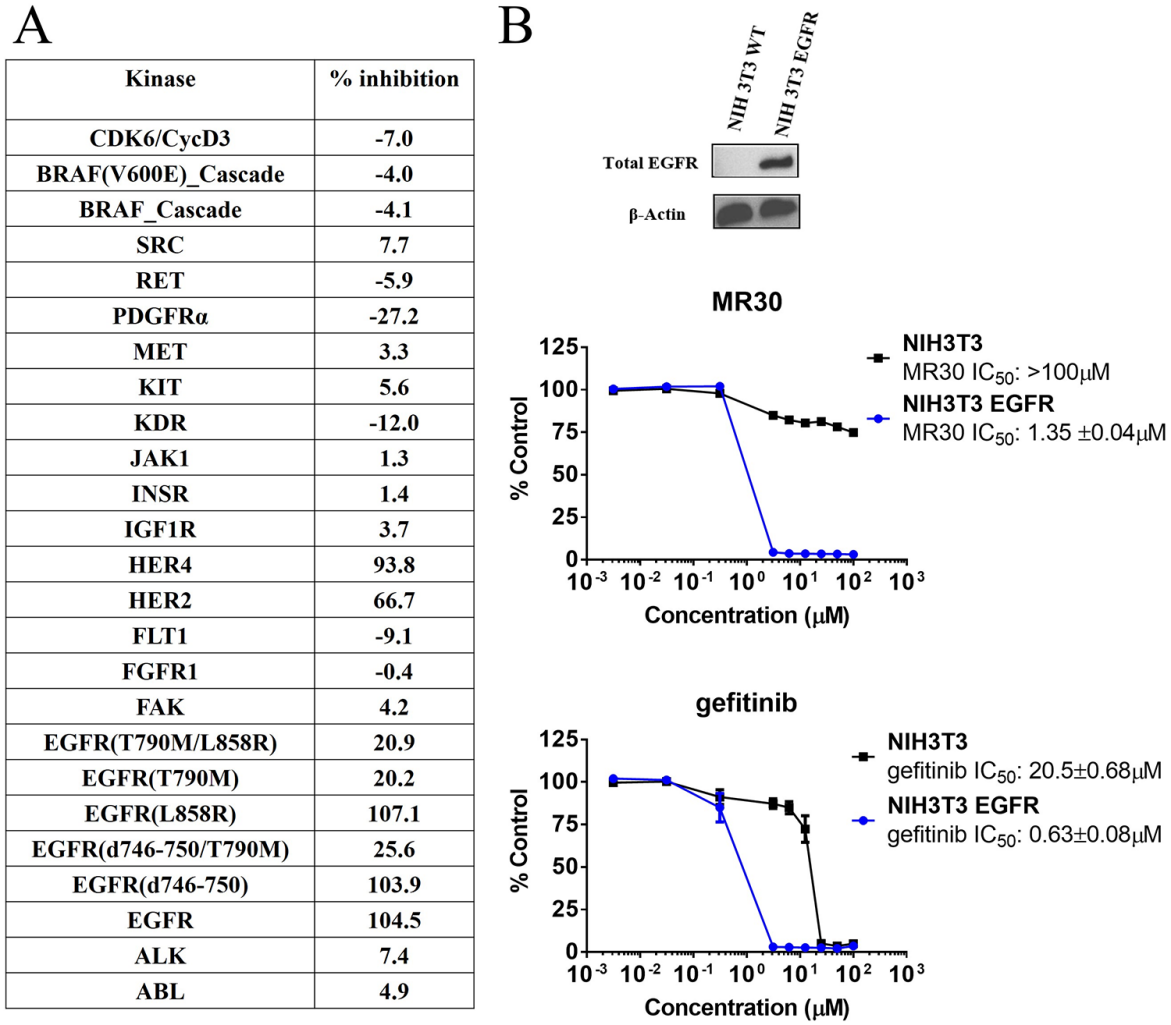
Preferential targeting of EGFR and its variants by MR30 in isolated and cell-based assays **(A)** Kinase inhibition of MR30 (500 nM) on 25 tyrosine and serine-threonine kinases (% inhibition). MR30 selectively inhibited kinases from HER family of proteins, including EGFR, EGFR (L858R, d746-750), HER2 and HER4. **(B)** Growth inhibition profile of MR30 and gefitinib in an isogenic model, NIH3T3 wild-type and NIH3T3 EGFR. Levels of EGFR for both cell lines are shown by western blot. Comparison of the selectivity of individual treatments (MR30, gefitinib). MR30 was more than 70-fold selective towards NIH3T3 EGFR compared to NIH3T3 wild-type cells. MR30 was more selective than the clinical EGFR inhibitor, gefitinib (32-fold selective) in this model.

In order to determine whether MR30 was selective for EGFR, we undertook a kinase profiling study using 25 tyrosine and serine-threonine kinases, evaluating the kinase inhibitory potency of MR30 at 500 nM. The choice of the kinases was based primarily on HER family (8 kinases) and other oncogenic kinases involved in the proliferation and stress response. Interestingly, MR30, selectively inhibited kinases from the HER family of proteins [e.g. EGFR wild-type, EGFR (L858R) and EGFR (d746-750)], as well as HER2 and HER4 showing little potency against other kinases of the panel (Figure 3A).

#### Selective targeting of EGFR expressing cells

In order to determine whether MR30 could selectively target EGFR-expressing cells, we evaluate its potency in isogenic cells expressing EGFR (NIH3T3 EGFR) in comparison with their wild-type counterpart NIH3T3. MR30 showed more than 70-fold selectivity towards NIH3T3 EGFR cells compared with the wild-type NIH3T3 cells. Furthermore, MR30 exhibited higher selectivity than the clinical EGFR inhibitor, gefitinib (32.5-fold selectivity) (Figure 3B).

#### MGMT targeting arm

**Figure 4:**
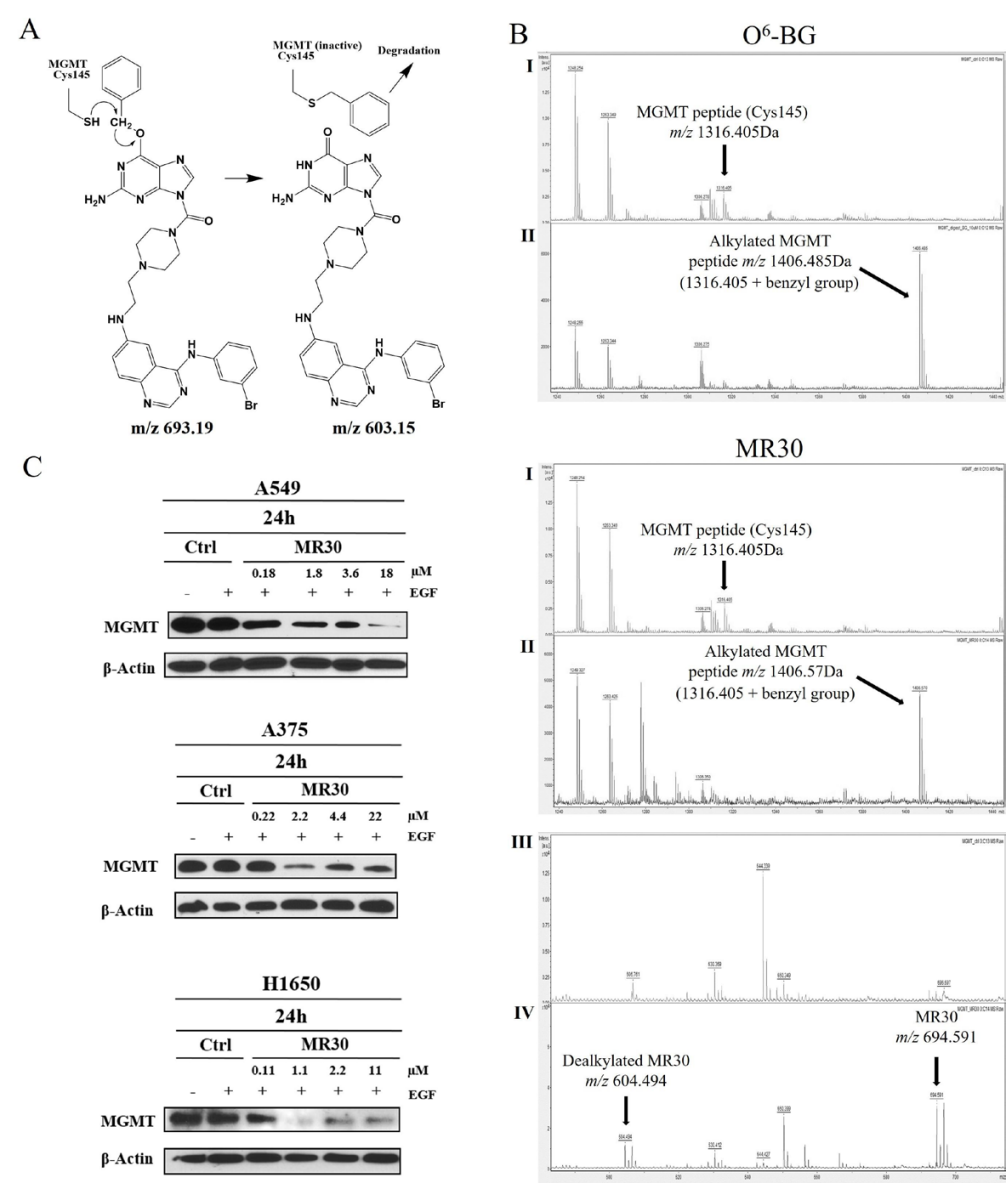
Molecular interactions and downregulation of MGMT by MR30 **(A)** Mechanism of the S-benzylation of Cys145 in MGMT through an SN2 reaction involving the SH group of MGMT with the benzyl CH_2_, with the dealkylated MR30 as a leaving group. **(B)** MALDI-TOF mass spectral analysis of tryptic peptides of unmodified MGMT (I) or MGMT incubated with the drug MR30 or O^6^-BG (II). S-benzylation of MGMT by MR30 or O^6^-BG (peptide *m/z* 1316.405+ benzyl group *m/z* 90.165) was observed at *m/z* 1406.57 or 1406.485, respectively. Mass of dealkylated MR30 was observed in the MALDI analysis at *m/z* 604.494 [(III (control), IV (treated)]. **(C)** Down-regulation of MGMT by MR30 was studied at 0.1, 1, 2 and 10x IC_50_ concentrations on A549, A375 and H1650 by western blot. MR30 down-regulated MGMT on all 3 cell lines in the 1-18 μM range.

The ability of MR30 to target MGMT was evaluated both *ex vivo* and in cells. As mentioned earlier, the mechanism underlying the inhibition of DNA repair activity of MGMT by O^6^-BG is the direct transfer of the benzyl group to Cys145 in an SN2 reaction leading to an S-benzylated enzyme (Figure 4A). Thus, *ex vivo*, we sought to determine whether the treatment of MGMT with MR30 would lead to the S-benzylation of Cys145 and in whole cells to its depletion.

#### *Ex vivo* analysis of MGMT-O^6^-BG interactions

The analysis of the S-benzylation of Cys145 was performed by matrix assisted laser desorption ionization-time of flight (MALDI-TOF) mass spectrometry of digested MGMT following treatment with MR30. A peak containing the cysteine residue (peptide *m/z* 1316.405) was detected in the control. Interestingly, when we treated MGMT with MR30 or O^6^-BG, we observed a mass shift (peptide *m/z* 1316.405 + benzyl group *m/z* 90.165) corresponding to the S-benzylated product (Figure 4B). Thus, like O^6^-BG, MR30 is capable of binding to MGMT and alkylate its Cys145, indicating that it can physically bind to the active site of MGMT. The transfer of the benzyl group was further confirmed by the detection of a 604.494 peak corresponding to a M-benzyl fragment of MR30 (see structure in Figure 4A).

#### Modulation of MGMT levels in whole cells

With the hypothesis that if indeed, MR30 can S-benzylate MGMT, the level of the latter should significantly decrease after treatment, we treated the cells with a dose range of MR30 and subsequently determine the levels of MGMT by western blotting. The results showed a dose-dependent down-regulation of the MGMT, 24 h post-treatment with strong inhibition in the 1-18 μM range (Figure 4C).

#### Mode of binding of MR30 to its dual targets

**Figure 5:**
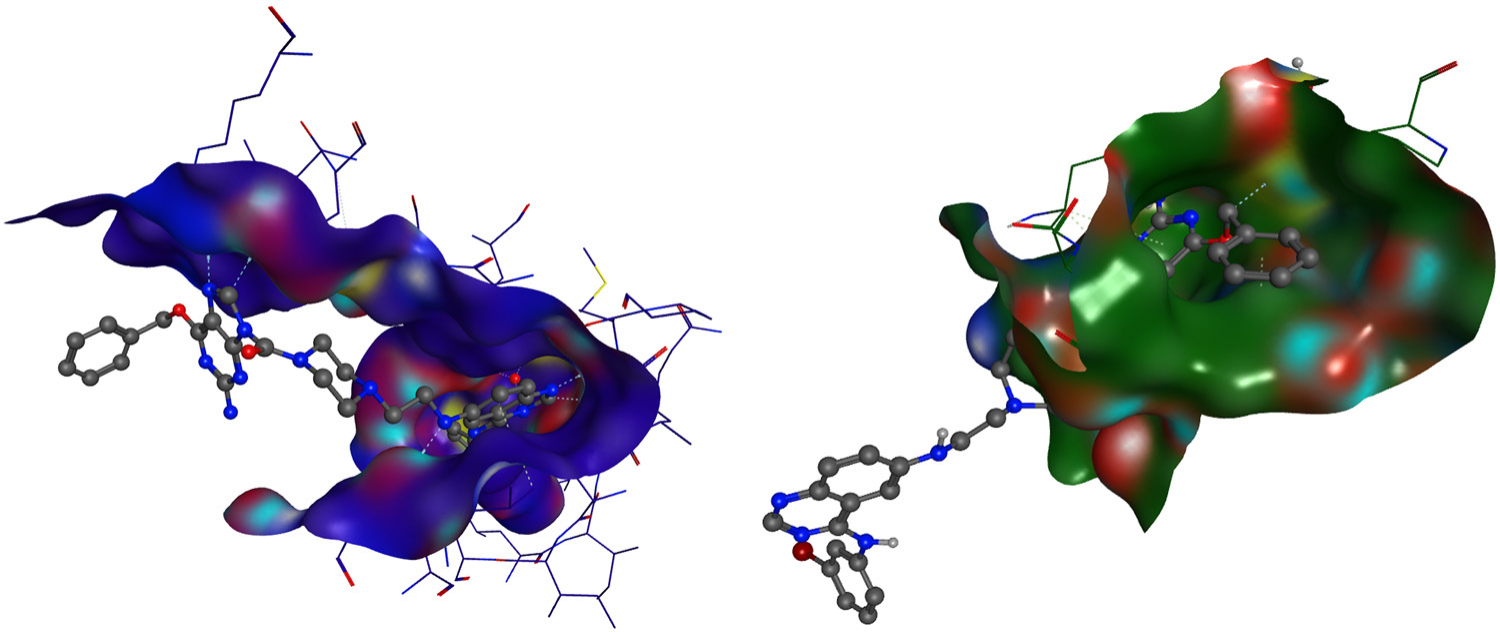
Molecular modeling of MR30 Molecular modeling of MR30 in EGFR kinase pocket (blue) and MGMT active site (green). The quinazoline moiety of MR30 is bound to the 1M17 structure (blue) in a pose analogous to erlotinib. In this pose, the MGMT portion and the urea linker of MR30 points out of the ATP binding pocket towards a solvent, allowing for conformational flexibility. The MGMT inhibitory portion of MR30 is in very similar position as O^6^-BG in 3KZZ (green), and maintained the same protein-ligand non-bonded interactions as O^6^-BG. The urea linker and the quinazoline portion of MR30 is solvent exposed and made no specific interactions with the MGMT active site.

Having shown that MR30 could directly alkylate the Cys145 in the active site of MGMT and inducing strong inhibition of EGFR phosphorylation and downstream signaling, we thought it of importance to determine how the chimera is bound to its dual targets. Molecular modeling was used to elucidate the binding mode of MR30 to EGFR or MGMT. MR30 was modeled in the EGFR kinase pocket using the 1M17 Protein Data Bank (PDB) structure as a starting point [35]. The quinazoline portion of bound erlotinib in 1M17 was used as a template to construct and minimize a bound structure of MR30. The quinazoline moiety of MR30 could bind to the 1M17 structure in a pose analogous to erlotinib. In this pose, the MGMT portion and the urea linker of MR30 points out of the ATP binding pocket towards the solvent, allowing for conformational flexibility. MR30 was also modeled in the active site of the MGMT enzyme using the PDB structure 3KZZ [36]. It was constructed and minimized in 3KZZ starting with the bound O^6^-BG ligand as the template. The MGMT inhibitory portion of MR30 was in the same position as O^6^-BG in 3KZZ, and maintained the same protein-ligand non-bonded interactions as O^6^-BG. The linker and the quinazoline portion of MR30 was solvent-exposed and made no specific interactions with the MGMT active site. A sample pose of MR30 modeled in EGFR ATP binding pocket and MGMT active site is showed in Figure 5.

### Comparison of potency of the dual targeted MR30 with a two-drug combination involving O^6^-BG + gefitinib

**Figure 6:**
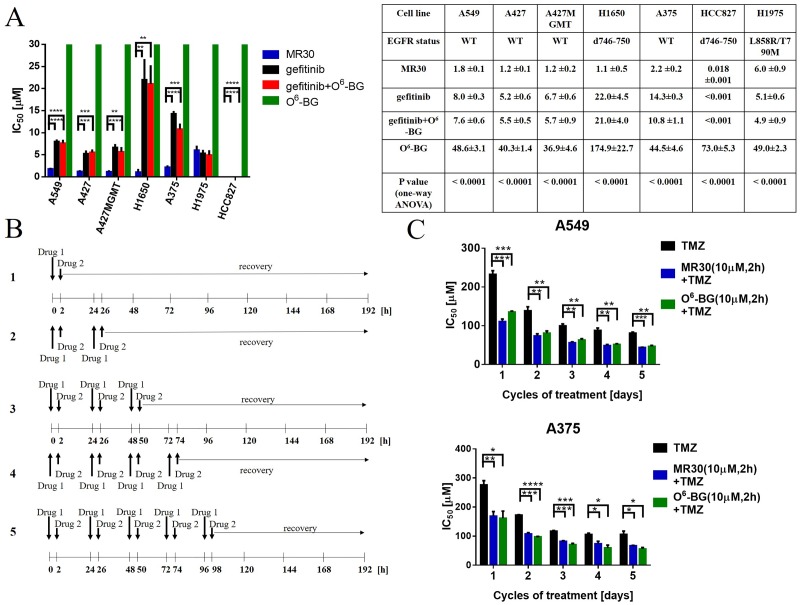
Effects of the dual targeting properties of MR30 on growth inhibition and sensitization of resistant cells to TMZ **(A)** Growth inhibition by MR30 in human tumour cells. IC_50_ values (μM, mean ±SEM) are means of at least three independent experiments. The drug combinations correspond to the equimolar combinations of gefitinib + O^6^-BG. MR30 showed stronger growth inhibition potency when compared to gefitinib, O^6^-BG and gefitinib + O^6^-BG in a panel of EGFR positive cell lines. A549, A375, A427, A427MGMT and H1650 cell lines were significantly more sensitive to treatment with MR30 in comparison to gefitinib alone or equimolar combination gefitinib + O^6^-BG. (^****^ p<0.0001, ^***^p<0.001, ^**^p<0.01). **(B)** Pulse exposure and sequence of administration of MR30 in combination with TMZ. Drug 1 (O^6^-BG, or MR30) was given for 2 h at a fix 10 μM dose. Thereafter, the medium was removed and replaced with Drug 2 (dose range of TMZ, 0.01- 400 μM) for 8 days. In sequence 2, a similar treatment was performed with the exception that a second Drug 1 and 2 treatment was administered 24 h after. In sequences 3-5, an additional treatment was incrementally added reaching a maximum of 5 repeated doses of Drug 1 and 2. **(C)** TMZ potentiation by MR30 or O^6^-BG was significantly (p<0.05) increased on MGMT positive cell lines A549 and A375. MGMT positive cells were pretreated with MR30 or O^6^-BG (both 10 μM) for 2 h. The drugs were washed out after 2 h and the cells treated with a dose range of TMZ. This sequence was performed for 1-5 cycles. Both MGMT inhibitors exhibited similar potentiation of TMZ. (^****^p<0.0001, ^***^p<0.001, ^**^p<0.01, ^*^p<0.05).

The dual potency of MR30 was compared with an equimolar combination of O^6^-BG and gefitinib. Thus, we first studied the activity profile of MR30, gefitinib, O^6^-BG in comparison with gefitinib + O^6^-BG on the panel of lung carcinoma and melanoma cell lines (Figure 6A). Surprisingly, MR30 alone showed significantly strong potency throughout the whole panel of cell lines. IC_50_ values in the panel of lung carcinoma cell lines were between 0.018 – 6.0 μM. MR30 showed its highest potency on HCC827 cells (EGFR d746-750) with an IC_50_ value of 0.018 ± 0.001 μM and its lowest potency on H1975 cells (EGFR T790M/L858R mutant) with an IC_50_ of 6.0 ± 0.9 μM. Importantly, MR30 showed significantly stronger potency than the clinical EGFR inhibitor gefitinib or the equimolar combination of EGFR and MGMT inhibitors (gefitinib + O^6^-BG) on 5 out of 7 EGFR expressing cancer cell lines (p<0.01). Indeed, A549, A375, A427, A427MGMT and H1650 cell lines were significantly more sensitive to the treatment with MR30 in comparison to gefitinib or the combination of gefitinib + O^6^-BG. Overall MR30 was a stronger antiproliferative agent than the combination of the single inhibitors.

### Studies on the potentiation of TMZ in established cell lines expressing EGFR and MGMT

MR30 was designed to potentiate the effect of TMZ in an EGFR-dependent manner. The strong potency of MR30 as a single agent translated into inhibitory concentrations that are below the levels reported for effective O^6^-BG-mediated potentiation of TMZ in MGMT-expressing cells. As previously described, many reports showed O^6^-BG-mediated TMZ potentiation in the 1-100 μM range [37–39]. Similarly, we observed that MR30 could down-regulate both MGMT and EGFR in the low micromolar range. Therefore, we investigated several combination models to simulate the pharmacological interactions of the dual targeted MR30 with TMZ.

First, MR30 was combined with TMZ at equimolar concentrations under continuous exposure. The results showed that the potency of the combination resembled that of MR30 alone (Supplementary Data 1). We believed that this significant potency was due to its EGFR targeting arm. Therefore, in order to effectively model the combination, we designed sequences of administration to attenuate the effects of its EGFR inhibitory potency. Our strategy was based on the putative reversible character of the EGFR inhibitory arm, the effect of which we planned to reduce through pulse exposures. As depicted in Figure 6B, five sequences were designed. First, Drug 1 (O^6^-BG, or MR30) was given for 2 h at a fix 10 μM dose. Thereafter, the medium was removed and replaced with a Drug 2 (dose range TMZ, 0.01- 400 μM) for 8 days. In sequence 2, a similar treatment was performed with the exception that a second Drug 1 and 2 treatment was administered 24 h after. In sequences 3-5, an additional treatment was incrementally added, reaching a maximum of 5 repeated doses of Drug 1 and 2. These repeated doses parallel the dosing schedule of TMZ in the clinic [40].

To perform this analysis, we focused on two, MGMT positive cell lines, with proven resistance to treatment with TMZ (Supplementary Data 2). The results showed that repeated doses of MR30 in combination with TMZ led to its significant potentiation in the cells (p<0.05-0.0001) (Figure 6C). Interestingly, the results were similar to those of TMZ + O^6^-BG administered in the same sequence, indicating that perhaps the attenuation of the EGFR potency of MR30 leads to an effect primarily induced by its O^6^-BG arm. Thus, these sequences of administration allowed to evidence the potentiation effect of MGMT targeting arm of MR30.

## DISCUSSION

Over the past two decades, a significant body of work has accumulated to demonstrate that the most common mechanism of resistance to genotoxic drugs is expression of DNA repair proteins capable of reversing the cytotoxic DNA lesions [13, 41–43]. Therapeutic approaches to reverse resistance focused on the discovery of DNA repair inhibitors capable of blocking the catalytic activity of the DNA repair enzymes [25, 44, 45]. Recently, tyrosine kinase receptor overexpression has begun to be associated to elevation of DNA repair protein levels and kinase inhibitors have been shown to sensitize cells to DNA lesions [1–7, 46, 47]. However, despite the validated role of many tyrosine kinases in tumour progression and drug resistance, combinations of DNA repair inhibitors with known kinase inhibitors are rarely studied. Here we explored the mechanisms and pharmacological interactions associated with the combination of inhibition of the most studied DNA repair protein MGMT and EGFR, a receptor tyrosine kinase to which is associated aggressive tumour progression and reduced drug sensitivity. Our analysis was performed using equimolar drug combinations in comparison with MR30, a single drug with the two EGFR-MGMT targeting arms and was further expanded to the study of the combination of the latter with the clinical drug TMZ.

The design of the molecule was based on two warheads associated with inhibition of EGFR and MGMT: the quinazoline and O^6^-BG, respectively. Importantly, the molecule was designed to include a water-soluble linker in order to favour bioavailability. More specifically, we exploited the N^9^-position of the O^6^-BG scaffold, which is tolerant of bulky substituents [48]. Likewise, the linker was branched to the 6-position of 4-phenylaminoquinazoline, which is tolerant of bulky substituents [49] to give a 694.6 MW molecule: MR30. The simulation of the mode of binding of MR30 using molecular modeling showed that the quinazoline moiety of MR30 was bound to the EGFR kinase pocket in a pose analogous to the clinical EGFR inhibitor, erlotinib. MR30 was also modeled in the active site of the MGMT enzyme. The MGMT inhibitory moiety of MR30 was in the same position as O^6^-BG in the MGMT active site, and maintained the same interactions as O^6^-BG. The model suggests that the combi-molecule cannot bind to the two targets simultaneously.

Experimental analyses of MR30 were performed in lung cancer cells representative of a disease for which EGFR inhibitors are indicated [32, 50] and melanoma cells deriving from a tumour type for which methyldiazonium generators such as dacarbazine and TMZ are indicated [34]. In addition to the kinase profiling that showed considerable selectivity for EGFR and other family members (HER2, HER4), the EGFR-targeting potency of MR30 in the cells was strong. Its ability to block HER2 and HER4 is an interesting property that may confer potency in HER2-dependent tumours. The dose dependent inhibition of EGFR autophosphorylation was associated with concomitant down-regulation of ERK1/2 and AKT phosphorylation. Interestingly, in the melanoma cell line, the EGFR phosphorylation was uncoupled with ERK1/2 phosphorylation, which we believe is due to the B-RAF (V600E) mutation in this cell line. However, AKT phosphorylation was strongly inhibited in these cells, suggesting that an important survival pathway was down-regulated by MR30 in these cells independently of the RAF-MAPK pathway status. These results are evidence of the strong EGFR inhibitory potency of MR30 and more importantly that the bulky water-soluble linker did not significantly affect its potency.

On the other hand, the S-benzylation of MGMT observed by MS analysis is a direct evidence of its ability to bind to the active site of MGMT. As depicted in Figure 4A, the mechanism of the S-benzylation of Cys145 can only occur through an SN2 reaction involving the SH group and the benzyl CH_2_, with the dealkylated guanine as a leaving group. Indeed, the mass corresponding to the dealkylated MR30 was seen in the MALDI analysis (*m/z* 604.494). Further whole cell analysis showed that indeed MR30 was able to induce a strong dose-dependent down-regulation of MGMT in the 3 cell lines at a relatively low concentration range (1-18 μM). This down-regulation may result from the digestion of the S-alkylated MGMT by protease enzyme in the cells.

It is important to note that we have demonstrated the role of each of the primary targets of MR30, with EGFR being a determinant for growth inhibition potency using the isogenic NIH3T3 cells. As indicated by the identical IC_50_ of MR30 in the A427/A427MGMT isogenic pair, MGMT does not play a role in growth inhibition. However, its dose-dependent depletion in the MGMT-expressing cells may be associated with its TMZ potentiation properties.

Having dissected the mechanisms of action of the two arms of the combi-molecule, we compared its potency with that of the two-drug combination mimicked by O^6^-BG and gefitinib: two drugs previously evaluated in the clinic [51, 52]. Interestingly, the chimeric nature of MR30 was proven superior to a two-drug combination at equimolar level. We believe that this may primarily be due to its strong EGFR inhibitory potency and perhaps little to its MGMT targeting arm. Indeed, the growth inhibitory potency of O^6^-BG in these cells was in the high micromolar IC_50_ range (36.9-174.9 μM). It is important to note that in the cell line with PTEN wild type and sensitive EGFR mutant (d746-750), the combi-molecule did not show superior potency when compared with gefitinib alone and the corresponding combination. These results are in agreement with the observation that gefitinib is a stronger EGFR inhibitor than MR30 and that it shows extremely strong potency in EGFR addicted cells (e.g NIH3T3 EGFR) [32]. Inversely, in H1650, A549, A427, A375 in which PTEN (H1650), K-RAS (A549, A427) or B-RAF (A375) are mutated, the combi-molecule is significantly more potent than gefitinib or corresponding combinations. Although the molecular mechanism underlying this observation remain to be elucidated, it clearly suggests that the new molecule MR30 could be a potent agent for the therapy of tumours in which the above genes (i.e. PTEN, K-RAS, B-RAF) are biomarkers for resistance to gefitinib.

The pleiotropic mechanism of action of MR30 indicated that as a single molecule, it could be a multitargeted potentiator of drugs that induce O^6^-methylguanine lesion. Thus, we studied its combination with TMZ under multiple modalities. At equimolar concentration combinations between MR30 and TMZ reflected the IC_50_ of MR30 alone, indicating that the potency of the EGFR targeting arm may have dominated the overall IC_50_. Given that the potentiation of TMZ requires higher micromolar concentrations of O^6^-BG, we designed washout experiments to attenuate the strong potency of the reversible EGFR inhibition of MR30. Interestingly, this led to significant enhancement of the potency of TMZ in cells with concomitant expression of EGFR and MGMT. Thus, we showed that at 10 μM, which is a concentration that is in the range required for O^6^-BG-mediated potentiation and S-benzylation of the Cys145 of MGMT, we could induce significant potentiation of TMZ in the same manner as O^6^-BG. Thus, these results demonstrate that indeed MR30 can act fully through its two major targeting arms.

**Figure 7:**
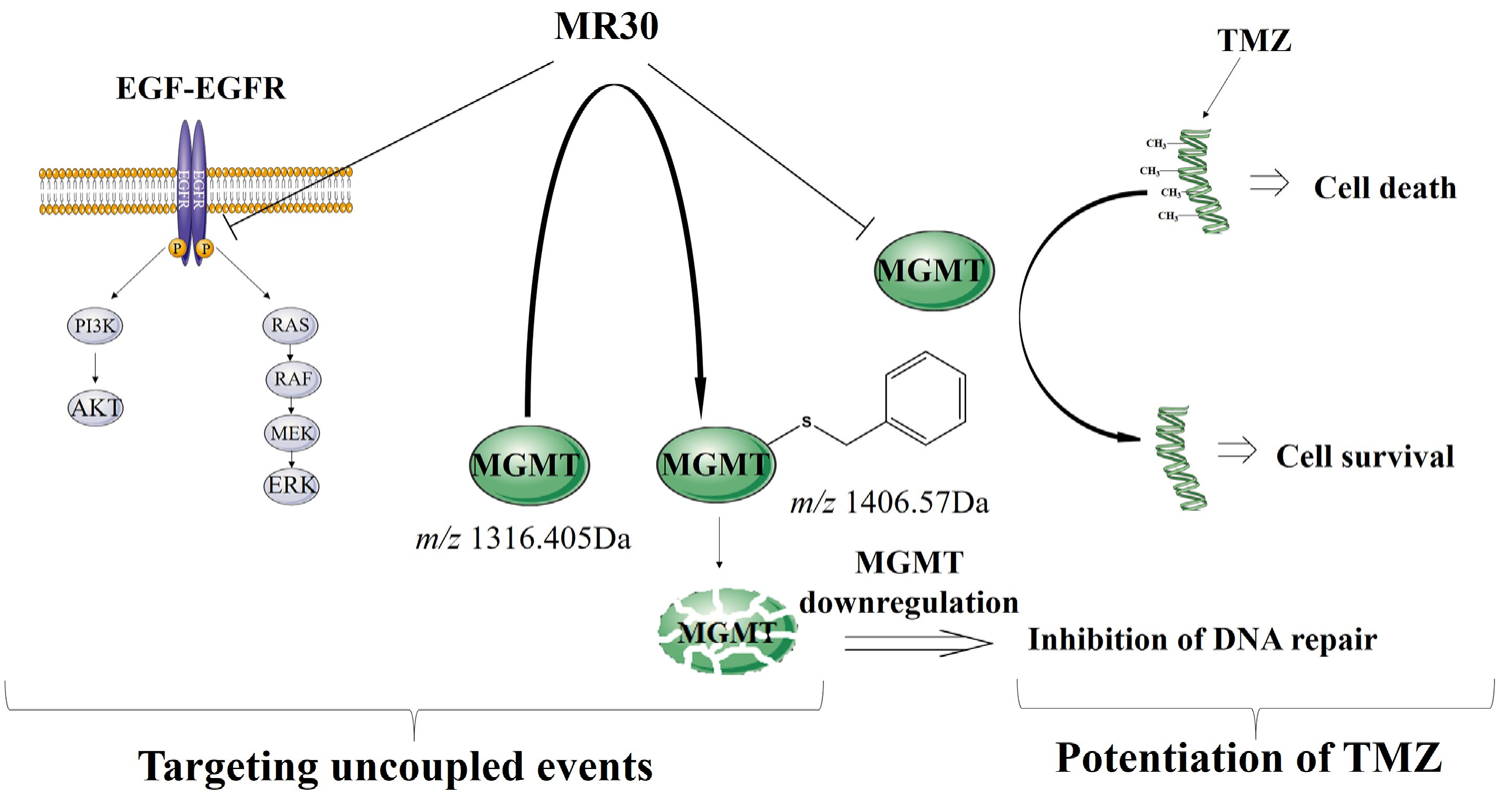
Schematic representation of the dual mechanism of action of MR30 Through its EGFR targeting arm MR30 can block RAF-MAPK-ERK1/2 and the PI3K-AKT pathways. Through its MGMT targeting arm, it can induce S-benzylation and subsequent down-regulation of MGMT and potentiate TMZ in tumour cells co-expressing EGFR and MGMT.

This work gave *prima facie* evidence of the feasibility of a bioactive EGFR-MGMT targeted molecule with superior potency when compared with a two-drug combination and ability to sensitize cells to TMZ. Importantly, the combi-molecule has proven potent in tumour cells with mutations associated with resistance to gefitinib. To our knowledge this is the first hybrid molecule shown to be able to down-regulate both EGFR-mediated signaling and MGMT. Thus, MR30 is a multifaceted molecule, which can be used under multiple contexts: (a) to exert selectivity for the HER family of oncogenes, (b) to induce potent growth inhibition in tumour cells with PTEN, K-RAS, B-RAF mutations and (c) to sensitize tumours that co-express EGFR and MGMT to TMZ. The pleiotropic activity of MR30 is summarized in Figure 7. Through its EGFR targeting arm it can block RAF-MAPK-ERK1/2 and the PI3K-AKT pathways. Through its MGMT targeting arm, it can induce S-benzylation and down-regulation of MGMT and potentiation of TMZ in tumour cells co-expressing EGFR and MGMT.

## MATERIALS AND METHODS

### Chemistry

^1^H NMR spectra were obtained on a Varian 300 MHz spectrometer and on a Bruker Ascend^TM^ NMR 400 MHz. Chemical shifts are given as δ values in parts per million (ppm) and referenced to the residual solvent proton peak. Mass spectrometry was performed by electrospray ionization (ESI) and analysis was performed at the Glen MUHC facility by Bruker Amazon SL spectrometer. Data are reported as *m/z* (intensity relative to base peak= 100). All chemicals were purchased from Sigma-Aldrich Canada Co. (Oakville, ON, Canada). RB10 and ZR2002 (4, Figure 1A) were previously synthesized in our lab [53, 54]. TMZ, O^6^-BG and gefitinib were purchased from Ark Pharm, Inc. (Arlington Heights, IL, USA).

#### 4-nitrophenyl-2-amino-6-(benzyloxy)-9H-purine-9-carboxylate

A solution of O^6^-BG (100 mg, 4.14×10^-1^ mM) in anhydrous pyridine (2 mL) was frozen in liquid nitrogen and chloroformate 1 added all at once. The mixture was stirred under argon for 2 h at 0°C and the reaction allowed to slowly reach room temperature. Thereafter, the solvent was evaporated to give carbamate 2 as a pure white solid (90 mg, 90%). ^1^H NMR (300 MHz, DMSO-d_6_) δ ppm 8.46 (s, 1H, ArH), δ ppm 8.4 (d, J= 9.2 Hz, 2H, ArH) δ ppm 7.75 (d, J= 9.2 Hz, 2H, ArH), δ ppm 7.53 (m, 2H, J = 8.2 Hz, ArH), δ ppm 7.44 – 7.37 (m, 3H, ArH), δ ppm 6.9 (s, 2H, NH_2_), δ ppm 5.5 (s, 2H, CH_2_).

#### (2-amino-6-(benzyloxy)-9H-purin-9-yl)(piperazin-1-yl) methanone

A solution of 2 (100 mg, 2.46×10^-1^ mM) in anhydrous DMF (1 mL) was added dropwise to a piperazine solution (9 equivalents). The mixture was stirred under argon for 4 h at room temperature. The reaction mixture was evaporated to give a crude product 3, which was purified by preparative TLC (silica plate, CH_2_Cl_2_/MeOH 80/20) to give pure urea 3 (25 mg, 25%). ^1^H NMR (300 MHz, DMSO-d_6_) δ ppm 8.04 (s, 1H, ArH), δ ppm 7.51 (d, J = 8.0 Hz, 2H, ArH), δ ppm 7.45 – 7.34 (m, 3H, ArH), δ ppm 6.72 (s, 2H, NH_2_), δ ppm 5.49 (s, 2H, CH_2_), δ ppm 3.32 – 3.29 (t, 4H, piperazine protons), δ ppm 2.86 – 2.78 (t, 4H, J = 4.6 Hz, piperazine protons). ESI-MS *m/z* 354.4 (MH^+^).

#### (2-amino-6-(benzyloxy)-9H-purin-9-yl)(4-(2-((4-((3-bromophenyl)amino)quinazolin-6-yl)amino)ethyl)piperazin-1-yl)methanone

To a solution of 4 (21 mg, 5.66×10^-2^ mM) in anhydrous DMF (500 μL) was added potassium iodide (2 eq). The reaction mixture was stirred for 24 h at room temperature. Thereafter, a solution of 3 (20 mg, 5.66×10^-2^ mM) with triethylamine (1.5 equivalent) was added. The reaction mixture was stirred for 10 days at 40°C and evaporated to give 5 (MR30), as a crude product, which was purified by preparative TLC (silica plate, CH_2_Cl_2_/MeOH 90/10). Compound 5 (MR30) was obtained as a pure yellow powder (5 mg, 25%). ^1^H NMR (400 MHz, DMSO-d6) δ ppm 9.37 (s, 1H), 8.38 (s, 1H, ArH), 8.17 (t, 1H, J = 1.9 Hz, ArH), 8.05 (s, 1H, ArH), 7.91 (d, 1H, J = 8.1 Hz, ArH), 7.57 – 7.49 (m, 3H, ArH), 7.43 – 7.31 (m, 5H, ArH), 7.26 (d, 1H, J = 7.9 Hz, ArH), 7.21 (d, 1H, J = 2.1 Hz, ArH), 6.68 (s, 2H, NH_2_), 6.07 (t, 1H, J = 4.9 Hz, NH), 5.50 (s, 2H, CH_2_), 3.51 (s, 4H, piperazine protons), 3.34 (q, 2H, J = 6.0 Hz, CH_2_). 2.71 (t, 2H, J = 6.4 Hz, CH_2_), 2.62 (s, 4H, piperazine protons). ESI-MS *m/z* 694.12 (MH^+^).

### Biology

#### Cell culture

A549 and A427 lung carcinoma cell lines were obtained from American Type Culture Collection (ATCC, Manassas, VA, USA). A427 MGMT lung carcinoma cell line was transfected in our laboratory by Dr. Ying Huang [12]. The H1975 and H1650 lung carcinoma cell lines were a generous gift from Dr. Kwok-kin Wong (Department of Medical Oncology, Dana-Farber Cancer Institute, Harvard Medical School, Boston, Massachusetts, USA). The NIH3T3 wild-type and EGFR (NIH3T3 EGFR) cells were a generous gift from Dr. Moulay Alaoui-Jamali (Lady Davis Institute, Jewish General Hospital, McGill University, Montreal, Quebec, Canada). A375 melanoma cell line was a generous gift from Dr. Richard Kremer (Department of Medicine, McGill University Health center, McGill University, Montreal, Quebec, Canada). HCC827 lung carcinoma cell line was a generous gift from Dr. Siham Sabri (Department of Medicine, McGill University Health center, McGill University, Montreal, Quebec, Canada). NIH3T3, NIH3T3 EGFR, A549, A427, A427MGMT, A375 cells were maintained in DMEM media. H1975, H1650, HCC827 cell lines were maintained in RPMI 1640 media. DMEM as well as RPMI 1640 media preparation was supplemented with 10% fetal bovine serum (FBS), 10 mM HEPES, 2 mM L-glutamine, gentamycin sulfate and fungizone (all reagents purchased from Wisent Inc., St-Bruno, QC, Canada) and were grown in a humidified incubator with 5% CO_2_ at 37°C. The media of each flask was changed when required and cell passaging was done between 85-95% confluence.

#### Drug treatment

MR30, TMZ, O^6^-BG, gefitinib and RB10 were dissolved in dimethylsulphoxide (DMSO) to form stock solutions and were further diluted in media as required.

#### *In vitro* growth inhibition assay

Cells were plated in 96-well plates (Corning, Corning, NY, USA) and subsequently allowed to attach for 24 h. Thereafter, they were treated with a wide range of drug concentrations to determine IC_50_ values of treatments. As for the single drugs and equimolar combination of drugs studies, cells were treated with MR30, TMZ, gefitinib, O^6^-BG or their respective equimolar combinations [(0.003125- 400 μM); 1% DMSO in DMEM]. Drug treatments were in triplicates for 5 days in the incubator (5% CO_2_, 37°C).

The potentiation study was carried out according to several sequences using two cell lines: A549 and A375. They were preincubated for 2 h with or without O^6^-BG or MR30 (10 μM; 1% DMSO in DMEM), the medium removed from all plates and replaced with TMZ [(0.01- 400 μM); 1% DMSO in DMEM]. A stock solution of TMZ was freshly prepared in DMSO. Plates were incubated for 8 days. Incubation with or without O^6^-BG or MR30 followed by treatment with TMZ was repeated at 24 h intervals. This was performed for a maximum of 5 cycles. Growth inhibition was determined using sulforhodamine B assay as described by Skehan *et al.* [55]. The results were analyzed using GraphPad Prism 6.0 (GraphPadSoftware, Inc., SanDiego, CA, USA) to derive a dose-response curve and the IC_50_ values. Every drug treatment on all cell lines was done in triplicates at least three times and the results represent an average of these independent experiments.

#### Kinase inhibition profiling of MR30

Kinase profiling was performed by Carna Biosciences, Inc. (Natick, MA, USA) website (http://www.carnabio.com/english/index.html) according to their standardized protocol. A total of 25 pre-selected kinases, including the entire HER family of tyrosine kinases and other serine-threonine kinases, were treated with MR30 at a single dose (500 nM). The competitive binding between the drug and ATP was conducted at the ATP concentration that is equal to Km for the individual kinase. For each kinase, the apparent Km for ATP was determined by a substrate (ATP)-velocity plot and the Michaelis–Menten equation.

#### MALDI-TOF mass spectral analysis

MR30 or O^6^-BG (10 μM) were incubated with MGMT (10 μg) (Cayman Chemical, Ann Arbor, MI, USA) in PBS for 3 h at 37°C. PBS was evaporated and 2 μg of MGMT were resuspended in ammonium bicarbonate (20 mM). MGMT was subsequently digested with trypsin (ratio MGMT: trypsin, 20:1) overnight at 37°C. The mixture was evaporated the following day and the resulting MGMT peptides were resuspended in 50/50 ACN/0.1%TFA. MALDI-TOF mass spectral analysis was performed on a Bruker UltrafleXtreme^TM^ (Bruker Daltonics, Bremen, Germany). Full scans of the peptide mixture from 700 to 3500 *m/z* and tandem mass spectral data of selected ions were collected with a-cyano-4-hydroxycinnamic acid as the matrix, as previously described [56].

#### *In vitro* kinase assay

The EGFR kinase assays was performed in 96-well plate (NuncMaxisorp) coated with PGT (poly L-glutamic acid L-tyrosine, 4:1, Sigma Aldrich, MO, USA) and incubated at 37°C for 48 h prior to use. PGT is the substrate, which is phosphorylated by EGFR (Enzo Life Sciences Inc, NY, USA, Signal Chem, Richmond, Canada) in the presence of ATP (50 μM). A dose range of drugs (1×10^-7^-10 μM, gefitinib or MR30) was added to compete with ATP to bind and inhibit the ATP-binding site of the EGFR kinase domain. To each well, 15 ng of EGFR (20 μg/ml) was added. The phosphorylated substrate was detected using an HRP-conjugated anti-phosphotyrosine antibody (Santa Cruz Biotechnology, Dallas, TX, USA). The signal was developed by the addition of 3, 3’, 5, 5’-tetramethylbenzidine peroxidase substrate (Kierkegaard and Perry Laboratories, Gaithersburg, MD, USA) and the colorimetric reaction was monitored at 450 nm using a microplate reader ELx808 (BioTek Instruments) [57]. The IC_50_ values were calculated using GraphPad Prism 6.0 (GraphPadSoftware, Inc., SanDiego, CA, USA). Each experiment was carried out twice in duplicate.

#### Molecular modeling

MR30 was modeled in the EGFR kinase pocket using erlotinib co-crystallized structure obtained from the Protein Data Bank (PDB) (code 1M17) downloaded from http://www.rcsb.org [35]. The quinazoline portion of bound erlotinib in 1M17 was used as a template to construct and minimize a bound structure of MR30. Minimizations were carried out in the MOE 2015.10 [58] software using the Amber10:EHT forcefield with R-Field electrostatics. MR30 was also modeled in the binding pocket of MGMT co-crystallized with O^6^-BG (PDB code: 3KZZ) [36]. MR30 was constructed and minimized in 3KZZ starting with the bound O^6^-BG as the template. The modeling was carried out in the MOE 2015.10 software using the Amber10:EHT forcefield and R-Field electrostatics for minimizations.

### Western blot

A549, A375, H1650, NIH3T3 and NIH3T3 EGFR cells were plated in 10% FBS containing media in 6-well plates and allowed to attach overnight (5% CO_2_, 37°C). The cells were rinsed with PBS 24 h later and starved overnight on addition of serum-free media. Thereafter, they were treated with different doses (0.1x, 1x, 2x and 10x IC_50_ concentrations for each cell line) of MR30 for 24 h, washed with PBS and stimulated with 50 ng/mL EGF for 30 min. Cells were washed, detached by scraping in cold lysis buffer 50 mM Tris-HCl pH 7.5; 150 mM NaCl; 1% Nonidet P-40, 1 mM EDTA; 5 mM NaF; 1 mM Na_3_VO_4_; protease inhibitor tablet (Roche Biochemicals, Laval, QC, Canada). Lysates were kept on ice for 30 min and collected after centrifugation at 13,000 rpm for 15 min at 4°C. The concentration of proteins was determined using the Bio-Rad protein assay kit (Bio-Rad laboratories, Hercules, CA, USA). Equal amounts of proteins were loaded, resolved on 10% SDS-PAGE and thereafter transferred to a polyvinylidene difluoride (PVDF) membrane (Milipore, Bedford, MA, USA). Membranes were blocked with 5% milk in TBST (20 mM Tris-HCl, 137 mM NaCl, 0.1% Tween 20) followed by incubation with phosphotyrosine antibodies such as phospho-EGFR, phospho-p44/42 MAPK, phospho-AKT (all purchased from Cell Signaling Technology, Danvers, MA, USA) in 5% milk, at 4°C overnight. The membranes were washed with TBST and incubated with respective secondary antibodies for 1 h and 30 min at room temperature in 5% milk in TBST. After incubation with antibodies against phosphotyrosines, the membranes were stripped using the Re-Blot Plus Strong buffer (EMD Millipore, Billerica, MA, USA) and probed for total EGFR (Santa Cruz Biotechnology, Dallas, TX, USA), total p44/42 MAPK, total AKT (both Cell Signaling Technology, Danvers, MA, USA) and total MGMT, Santa Cruz Biotechnology, Dallas, TX, USA) with the corresponding antibodies along with β-actin (Cell Signaling Technology, Danvers, MA, USA) antibody. For the detection of EGFR levels in the NIH3T3 isogenic pair of cell lines, no EGF-stimulation was performed. Immunoblot bands were visualized using ECL kit and enhanced chemiluminescence system (ThermoFisher Scientific, Waltham, MA, USA).

### Fluorescence imaging of MR30 in A549 cells

Using a Tecan Infinite M200PRO plate reader, scanning of an ethanol solution of MR30 showed that its maximum emission peak was 458 nm at a 280 nm excitation. Therefore, fluorescence imaging was performed at 458 nm. Briefly, A549 cells were plated at 70% confluence in petri dishes, allowed to adhere overnight, and treated with 25 μM of MR30 or RB10 for 3 h. At the indicated time point, cells were fixed with cold methanol at -20°C for 5 minutes. The cells were subsequently washed with PBS and nuclei stained with propidium iodide (PI) (5 μg/mL) (ThermoFisher Scientific, Waltham, MA, USA). Images were captured with a Zeiss LSM 780 laser scanning confocal microscope.

### Statistical significance

Statistical significance for *in vitro* assays was performed using the unpaired, two-tailed student t-test and one-way ANOVA. Significance was claimed with p<0.05. GraphPad Prism 6.0 (GraphPadSoftware, Inc., SanDiego, CA, USA) was used for statistical analysis.

## SUPPLEMENTARY MATERIALS FIGURES AND TABLES



## Abbreviations

Bax: BCL2 associated X protein
DMSO: dimethylsulphoxide
EGF: epidermal growth factor
EGFR: epidermal growth factor receptor
ERK1/2: extracellular signal-regulated kinase 1/2
ESI: electrospray ionization
ERCC1: excision repair cross-complementation group 1
FBS: fetal bovine serum
HER: human epidermal growth factor receptor
MALDI-TOF MS: matrix assisted laser desorption ionization-time of flight mass spectrometry
MAPK: mitogen-activated protein kinase
MGMT: O^6^-methylguanine-DNA methyltransferase
NMR: nuclear magnetic resonance
O^6^-BG: O^6^-benzylguanine
PDB: Protein Data Bank
PGT: poly L-glutamic acid L-tyrosine
PI3K: phosphatidylinositol-3 kinase
ppm: parts per million
PTEN: phosphatase and tensin homolog
PVDF: polyvinylidene difluoride
TLC: thin-layer chromatography
TMZ: temozolomide
XRCC1: X-ray repair cross-complementing protein 1
