# Supplementary material for: Molecular analysis of the dual targeting of the epidermal growth factor receptor and the O6-methylguanine-DNA methyltransferase with a double arm hybrid molecule
Source: Oncotarget. 2018 Oct 12;9(80):35041–55. doi: 10.18632/oncotarget.25120 (PMC6205551; doi:10.18632/oncotarget.25120)
Supplement: Supplementary file 1 [file oncotarget-09-35041-s001.pdf]

Molecular analysis of the dual targeting of the epidermal growth factor receptor and the O^6-methylguanine-DNA methyltransferase with a double arm hybrid molecule

SUPPLEMENTARY MATERIALS

Supplementary Data 1: Comparison between MR30 with its corresponding combination with TMZ

Cell line	MR30	MR30+TMZ
**A549**	**1.8±0.1**	**1.6±0.3**
**A427**	**1.2±0.1**	**1.0±0.003**
**A427MGMT**	**1.2±0.2**	**1.4±0.2**
**H1650**	**1.1±0.5**	**1.8±0.5**
**A375**	**2.2±0.2**	**2.2±0.2**
**HCC827**	**0.018±0.001**	**0.014±0.001**
**H1975**	**6.0±0.9**	**5.9±0.9**

Growth inhibitory potency of MR30 compared to equimolar combination of MR30+TMZ (IC_{50} values (µM), mean ±SEM) under continuous exposure. The potency of the combination resembles that of MR30 alone.

Supplementary Data 2: Evidence of TMZ resistance of our panel of cell lines

Cell line	MGMT status	TMZ
**A549**	+	**283 ±24**
**A427**	**-**	**15.5 ±5**
**A427 MGMT**	+	**253 ±28**
**H1650**	+	**>400**
**A375**	+	**222 ±15**
**HCC827**	+	**373 ±27**
**H1975**	+	**>400**

Growth inhibitory potency of TMZ (IC_{50} values (μM), mean ±SEM) on a panel of tumour cell lines. MGMT positive cells exhibits resistance to treatment with alkylating agent TMZ.
